# Host manipulation by parasites David P. Hughes, Jacques Brodeur, and Frédéric Thomas, eds Oxford University Press, UK

**DOI:** 10.1111/eva.12062

**Published:** 2013-05-15

**Authors:** Conrad Cloutier

**Affiliations:** ^1^ Département de Biologie Université Laval Laval QC Canada

The book is a beautiful combination of expert thinking and knowledge on parasites exploiting multicellular host individuals, populations, societies and communities, as their ecosystem. The book comprises eleven chapters by expert behavioural ecologists as main authors, each one appended by an afterword from a nonbehavioural ecologist, a briefer reflection on the main authors' treatment. Reading this book can entertain any biologist's curiosity for the ubiquitous phenomenon of parasitism, and especially animal behaviour manipulation by microbial or animal parasites. I would recommend it or at least parts of it as reading to undergraduates taking introductory zoology, or as a main source for graduates in ecology, evolutionary biology, animal parasitology, medical and veterinary sciences, and biodiversity conservation.

In the book foreword, Dawkins reminds us of his *extended phenotype* paradigm, which sets the host organism from the evolutionary viewpoint of the parasite: the parasitized host phenotype is ‘bended to a direction hostile to the host's own genes’. But hostility varies widely across parasitic phenomena from negligible or benign, to stressful but tolerable (reduced growth and reproduction), and in extreme cases to host suppression either reproductively or completely (death) thus freeing the parasite into a dispersal stage. Parasitism is most intricate for multi‐host parasites with complex cycles whose final (reproductively) host is a predator. The life style of Dawkins' *Verticobacter* seems to match that of *Toxoplasma gondii* in its parasitic relation to humans (see Chap 10, and below). As opposed to its lifestyle in primary host rodents, *Toxoplasma* in humans seems not to trouble with sex, its persistence being delegated to our viviparous reproduction ensuring direct continuity between mother and infant as successive hosts in the parasite's life.

In Chapter 1, Moore tells us an informative story of how and when traditional parasitology recently became ‘cool’ or popular. Parasites at a node point of their cycle and making their host especially appealing to a predator were thought of as manipulators well before the ‘cool age’ (Moore cites Siebold 1853 on mode of transfer of *Leuchochloridium* to its next host). Like for other organisms, there is no goal or direction in parasite evolution. Thus, there is no support for benign parasites consistently emerging from brutal ancestors. A pattern with more support is that relatively small parasites, which reproduce in the host, are selected more rapidly and evolve more often to sympatric speciation. Compilations by Moore indicate that parasites and their altered hosts became popular in the late 1900's, following the birth of many ecology journals. Moore reviews altered host behaviours that do not benefit parasites, such as vertebrate avoidance/defence against biting flies, self‐medication and grooming, anorexia, sleep and ‘depression’ redirecting host resources to combat pathogens and ectotherms’ behavioural fever with similar effects. Moore says we must thank behavioural ecologists for revealing the ‘cool side’ of parasites that make their hosts behave strangely. Part of Moore's exposé tells us of the many scientist women who have been leaders in parasitology, including ‘cool’ aspects such as induced host suicide as a key event in parasite transmission to predators, and the role of parasitism in sexual conflict (mate choice).

Afterword author Alcock also praises the contribution of behavioural ecology to parasitology, pointing out the very productive and influential idea that female mate choice (in e.g. birds), may have evolved because of variable susceptibility to parasites among males.

In reviewing evolutionary routes to manipulation in Chapter 2, Thomas, Rigaud and Brodeur consider two scenarios of parasitized host behaviour evolution, that is, parasite‐driven (most studies) and host‐driven, for example compensatory foraging or mating. As parasites first evolved from free‐living ancestors, the second scenario seems more likely. Ancestral parasites were probably surviving and exploiting abundant resources in the digestive, aerial, or coelomic body cavities of accidental hosts. As pointed out, enhanced naturally iterative host behaviours such as sex and foraging can benefit fitness of both host and parasite, as demand‐driven compensating behaviours. When even brief parasitism allows parasite survival and growth or reproduction, traits favouring parasitism durability and fixation, including host manipulative traits, are strongly selected. Interactive manipulation should persist as a ‘cool’ topic in parasitology, as parasitized hosts are relatively easy to observe, although they are in fact complex evolving units with intraspecific variability arising at multiple levels between genomes, species stages, sexes and morphs of the hosts and parasites.

Afterword author Stearns expectedly addresses manipulation within the context of life cycle evolution. A panoply of evolutionary ideas can emerge when our thinking switches from individual organisms to interactions involving not only the individual with the other sex, family or group members, stages and morphs within a species, but also with heterospecific symbionts, mutualists, pathobionts and parasites. The close associations they form with the host involve each one's phenotype (and genotype) and range more or less widely on the symbiosis spectrum. Each one constitutes a regular and reliable external feature of the other's environment, which determines their fitness. Stearns suggests that host manipulation by a parasite evolves when a previously dispersive stage establishes in a potential next host, to which it then ‘delegates some of its fitness traits’. Coevolution eventually eliminates traces of the original dynamics, leaving us with an egg‐chicken puzzle.

In Chapter 3, Adamo reaches into the fine strings of behavioural manipulation, with examples from the famous or infamous ‘puppet masters’, such as brain cysts‐forming *Toxoplasma* manipulating rodents into being easier prey for their cat enemies. Even with good reasons for behavioural alterations having a parasitic or symbiotic cause, we should never be content until a clear biological mechanism has been identified and properly tested. If a nematomorph worm indeed has the tools to drive the oriented move of a cricket towards a body of water and then to take a bath so that the worm finally reaches its aquatic reproductive milieu, then the mechanical linkages between the worm driver and the cricket vehicle must be described and shown to explain the original detour (Ponton et al. 2011). The ‘explanation’ of the fitness advantage for the worm is not satisfactory, convincing and conclusive. We are still left with mostly indirect and obscure mechanisms at work between the two animals. One might suggest for instance that host ‘hygienic bathing’ possibly explains the facts more parsimoniously than parasite manipulation. The cricket, which may survive, rids itself of its wormy ‘master’ while entering the water. Adamo exposes that infection‐related immune modulation (via e.g. cytokines) alters vertebrate neural function in multiple and complex ways, inducing sickness, fatigue and irritability, a system that parasites could evidently exploit, if not manipulate. The author briefly summarizes (Table 3.2, p.45) known effects of parasites in terms of neural anatomy, chemicals and immune factors, giving us a rather unconvincing picture of how parasites could do it. Parasites do not seem to target specific neural sites and even brain cysts lodge in places that are not involved in complex behaviour. Parasites may rely on analogue chemicals of their own, acting on host behaviour status or mode with biogenic amines (dopamine, serotonin), thus acting on host neuromodulator pathways, but this still seems far from effectors of sensory‐driven oriented locomotion.

Afterword author Robinson reminds us that behaviours are either instinctive or experiential. If host neural effectors predictably change with infection or parasitism, then we could learn about behaviour manipulation by noxious symbionts from the induced changes. Appropriate behaviours occur as responses to stimuli at the right time and place. If behaviours evolved to be rewarding, then Robinson asks how do parasites cause host suicide? One might suggest that cell apoptosis in multicellular organisms could help us understand. Apoptosis occurs as a genetically built‐in defence in immune reactions or when cells become ineluctably damaged by disease or noxious agents (Norbury and Hickson 2001). At the supra organismic level, individual worker suicide could possibly evolve to the same ultimate colony ‘curing’ effect as cellular apoptosis, as an evolutionary response to consistent and reliable experiences of attack by noxious invaders.

In Chapter 4 on the behavioural ecology of parasitized hosts, Roitberg uses the power and freedom (beyond that of experimental work) of modelling to look into the question of host manipulation from a holistic perspective. Focus is on foraging decisions of hosts experiencing internal (e.g. energy level, growth) and environmental (stochastic) constraints, with or without parasitism and manipulative behaviour. Stochastic, dynamic state variable models can address host manipulation in all its useful complexity, the host role being taken here for convenience by a caterpillar using its natural foraging repertoire. The interplay of environmental heterogeneity and state dependency of both host and parasite is examined in a behavioural ecology context. The caterpillar can alter its use of nutrients and its feeding, foraging and dispersal rates either under its own control or the parasite control.

Afterword author Dubois praises the focus on variation between host individuals, that is, the basis of evolutionary change, in examining fitness costs and benefits, to determine whether host behaviour alteration benefits host or parasite. On commonly depressed parasitized host feeding, Dubois comments that because immunity and growth can be traded off in a host, variation on the optimal behavioural landscape can help us answer the key question, that is, who is causing host behaviour alteration?

In chapter 5 on plants manipulation, Mescher focuses on the hospitality (or inhospitality) of higher plants to parasites such as aphids, caterpillars and fungi, as manipulators. Plants are evolutionarily experienced multicellular hosts to many organisms, especially insects. The ‘green lineage’ as a eukaryotic main branch evolved as autotrophs via hospitality to photosynthetic cyanobacteria, residing today as permanent organites in all plant cells, such as the chloroplasts. Mesher points out that long‐lived vascular plants, especially trees, literally define natural habitats, supporting millions of microbial and small multicellular consumers and residents. Plant individuals or clones as hosts to communities of smaller organisms are exceptionally reliable, both spatially and temporally. In contrast to most multicellular animals, plants do not move and their ‘behaviours’ generally occur at rates orders of magnitude slower than animals. Focus is on plant phenotypes that specifically improve plant quality for insects, for example, growth pattern, providing shelters such as galls, fuel for flight as nectar or take‐home fast food as pollen, and that are shaped by selection on herbivore genes. These plant traits are the insect's extended phenotype *sensu* Dawkins, that is, they are plant traits adaptive to the insects they house (Danks 2002). Herbivore insects as plant parasites can thus become ecosystem engineers (Jones et al. 1997), with effects extending to the community level (Agrawal et al. 2012).

Plant galls and insects inducing them have long been notorious as a case of heterospecific manipulation. Galls as plant structures produced ‘for the good of another species’ is an observation Darwin wrote he could not explain by Natural Selection. Galler insects can even induce extra floral nectaries, thus recruiting ants as bodyguards. Phylogenies show that related insect gallers induce similar galls on different plants, and gallers produce specific galls on the same plant. Gall induction is known to be under control of galler genes, which remains to be seen in host behaviour manipulation by parasites. Mesher reviews other fascinating aspects of plant manipulation by parasites. An interesting aspect having little appeal for ecologists interested in overt behaviour is plant defence signalling governed by phytohormones (salicylic acid, jasmonates, ethylene). Antagonisms, chemical trickery and cross talk are key interactions in the quiet and silent life of manipulators such as fungi and phloem feeders such as aphids and whiteflies. Sessile life in plants led to evolving original sexual reproduction and offspring dispersal, for example, by manipulating small visitors and especially insects with a taste for sweets or deceitful sex (Bohman et al. 2012) as pollinators, with repercussions in these traits being exploited by plant parasites. Many plant pathogenic fungi (e.g. *Puccinia*,* Uromyces*,* Ustilago*) manipulate flower appearance to pollinators (pseudo‐flower phenotypes), which fungi use for outcrossing.

To afterword author Jordano, plant manipulation has evolved way beyond reciprocal interactions, to third party players as top manipulators (see Fig. 5.3, p 93). Common reliable interactions based on relatively large plants and their multiple insect exploiters are clearly the targets of evolutionary usurpation. Behaviour is overt in animal hosts, but in host plants, its manipulation has more diverse and easily overlooked effects. Plants commonly house multiple ‘partners’ from several trophic levels. We need to better describe and understand interactions that the higher plants set a grand stage for.

In Chapter 6, Langmore and Spottiswoode focus on manipulative visual mimicry in avian brood parasites, where costs to hosts often vary with host defences. The arms races hypothesis is examined stepwise from parasite egg laying, to egg incubation and to chick feeding by the host. Special attention is focused on traits of parasitic chicks exploiting host parental communication and care and contrasting generalist vs. specialist brood parasites. Host egg mimicry has been known for a long time in cuckoos and has evolved independently at least six times providing strong support for its adaptiveness to brood parasites. Experimental studies with spectrophotometric and visual modelling techniques have considerably refined work. The most discriminant hosts are those challenged by cuckoos laying eggs that are the most mimetic with respect to host visual perception, egg colour and patterns. Langmore and Spottiwoode compiled over 20 experimental studies (Table 6.6 p. 98) of brood parasite egg rejection. Alternatives to host rejection as evolutionary pressure on egg mimicry include competition for parental care and predation. Also considered are counter‐defences based on adaptive variation of host egg patterns, clutch polymorphisms and the possibility that parasitic birds learn to choose host egg clutches adaptively. Visual trickery other than simple mimicry has also been studied. Egg rejection may be avoided by crypsis analogous to chemical ‘insignificance’ in social insect parasites or by exploiting natural colour preferences in host birds, such as for plumage colour. Mimicry of host chicks is less frequent, either because evolution of host defences against parasitic solicitation of parental care is biologically constrained or has not been sufficiently investigated. But striking chick mimicry occurs in cuckoos, based on colour, chick down clothing and mouth gape and associated begging. Parasitic chicks must boldly stimulate parental feeding, a different need than simply escaping early rejection. They often grow faster than host chicks. *Vidua* finches are a group of host specific parasites whose chicks closely match their host mouth gape pattern. Competition with host chicks might lead to evolution of exaggerated solicitation signals, that is, irresistible gape patterns and more frequent or intense begging calls. Generalist bird parasites can adapt behaviourally to different hosts based on learning to mimic host begging calls and morphologically by producing ‘average’ egg or chick appearance. The authors adhere to distinguishing between arms race coevolution of mimicry and discrimination and evolutionary fine‐tuning of host care manipulation by growing chicks. This fine‐tuning process would not typically lead to evolution of reciprocal counter adaptations.

Afterword author Edwards starts by asking basic evolutionary biology questions about how natural selection, in the context of population genetics, may have led to quasi‐perfect mimicry such as leaf‐mimic frogs or insects, or cuckoos closely resembling hawks. Among brood parasites, these cuckoos would represent a less common case of Batesian rather than Mullerian mimicry. Molecular methods recently used to test the arms race hypothesis for egg mimicry in cuckoos bring genetic evidence into the picture, but the phylo‐geographic patterns suggest that host races, known as *gentes* in bird brood parasites, have evolved rapidly and probably form unstable associations subject to obliteration. Edwards stresses that gene mapping and sequencing used in the context of race for race, if not gene for gene stepwise evolution, will be relevant to the study of these systems in birds. Genomics in brood parasitism could do more than resolve the traditional sources of adaptation, that is, origins, ontogeny, mechanisms and functions.

In Chapter 7, Miller and Schneider discuss microbial symbionts focussing on *Wolbachia* bacteria as advanced manipulators of arthropod hosts, but also briefly discuss insect viruses and ectoparasitic mites of beetles. *Wolbachia* is an obligate vertically transmitted endosymbiont of insects, crustaceans and mites, in addition to nematodes and trematodes. *Wolbachia* is best known to manipulate host reproduction, especially behaviour facilitating its transmission across host generations (see Table 7.1 p. 120). Three major phenotypes of W^+^ hosts are *cytoplasmic incompatibility* (suppression of noncarrier zygotes, especially males), male *feminization* (genetic males with female anatomy and behaviour) causing sex ratio distortions and *thelitokous parthenogenesis*. Male hosts are useless as *Wolbachia* generally propagates as cytoplasmic voyageurs via female gametes and zygotes. *Wolbachia* manipulators affect host defence and immunity, host mate choice based on olfactory cues and bias host reproductive fitness towards or exclusive to W^+^ hosts. These effects often have direct evolutionary consequences via reproductive isolation potentially leading to speciation. Male feminization has been studied in various hosts including *Armadillo* isopods, Lepidoptera and *Drosophila*.

Afterword author Ehrman says he was directly involved in the discovery of the *paulistorum* species complex of *Drosophila* as Dobsjansky's PhD student. For a schematic view of the symbionts involvement in this cryptic species complex, and the role of *Wolbachia* voyageurs as determinants, see Tables 7.2 and 7.3, p. 129. In discussing reproduction isolating mechanisms at work in this ‘superspecies’ (p. 139), Ehrman writes it is still unclear to him where *Wolbachia* belongs in the large mutualist–parasite continuum.

In Chapter 8, Hughes focuses on manipulation of social insect colonies as superorganisms*,* whose collective phenotype results from specialization of individuals over reproductive and alternative functions, such as in termites, ants, and social wasps and bees (eusocial insects). Referring to ant colonies as superorganisms goes back to Wheeler (Wheeler 1911), but the idea has frequently met with inherent difficulties. In particular, discerning the appropriate level of selection (colony vs. individuals and their genes), the uncertain functional unity of the group and conflicts over resource allocation to genders (sex ratio, not applicable in termites) or morphs, for example, workers vs. males and queens among progeny raised by the colony. Hughes stresses the additional conflicts that parasites introduce in superorganisms by making colony members cheaters, as vehicles of parasite genes. But natural selection does not act directly on the cheaters' behaviour, as a social insect colony can modify its worker ratio in response to parasitism, a form of homoeostasis at the superorganism level. Although not all behavioural changes induced by social parasites are parasite‐adaptive (e.g. hygienic grooming), those that clearly are adaptive constitute neat examples of how the activity (or inactivity) of an individual host can be controlled by parasites. In ants for example, parasitized workers may tend to stay home instead of foraging, thus limiting the inherent risks of leaving the nest. Conversely, many trophically transmitted parasites (several invertebrates, fungi) force workers to go out to free a parasite or make it available to a next host, a predator of the former host.

To many biologists, the most striking parasites of social insects are those entering nests and living among social insect colonies. These so‐called social parasites include ants, bees, beetles, caterpillars, flies, molluscs and mites revealing the ecological and evolutionary opportunity that large and stable social insect nests represent. Social parasites have no permanent host contact and are not parasites *sensu stricto*, but reside in colonies as independent wanderers, stealers and consumers of the brood and/or the food resources (e.g. honey, attine garden fungi), or as microbial symbionts in attine ants. Host manipulations include various levels of host mimicry in form, odour and behaviour based on close contact signalling and workers corruption with irresistible or addictive secretions as currency. Hughes stresses that further study of the parasites of superorganisms requires a socio‐parasitological mindset, aiming to address the additive and interactive effects of social parasites and discover thresholds at which the loss of colony members translate into feedback into the evolution of colony defence.

In the afterword, Hölldobler discusses applying the superorganism concept to ants. Ants exhibit a range of cohesion from highly competitive to tightly organized groups, such that the cohesion level at which superorganismic traits emerge must be subjective. Poneromorph ants should not be called superorganisms. Interestingly, they have few or no social parasites, in contrast with true ants, for example *Formica* and the attines, which house communities of parasites. Members of superorganisms have self‐recognition mechanisms such as chemical codes or behavioural stereotypes, which social parasites have to break to be tolerated, and especially to release worker care behaviour.

In Chapter 9, Lafferty and Kuris discuss conditions for behavioural manipulation to have ecological and evolutionary impacts. This will depend on the incidence of manipulator parasites and the ecological importance of their hosts. Like others in the book, they stress the distinction between adaptiveness to the parasite vs. adaptiveness to the host. Altered behaviours benefiting the host and having community effects are well known, for example fish on coral reefs relying on cleaner wrasses to control ectoparasites. Demonstrating ecological impacts have relied on modelling the intensity of such effects (prevalence) and examining statistical properties of parasitism distribution in host populations (incidence). By taking over host ecological identity, parasitic castrators and parasitoids can modify host competition and susceptibility to predation, depending on the differential between hosts and nonhosts. Among the most cited and popular cases, crickets hosting a nematomorph are twenty times more likely to fall prey to charr in Japan streams. Ecologically, the worms move the energy collected on land by the cricket to stream aquatic habitats. Tapeworms forming weakening hydatid cysts in moose and using wolf as final host can regulate moose density, and indirectly protect the forest on Isle Royale. A similar ecosystemic story is documented for trematode parasites of cockle, which are clams living on Australian mudflats. Trematodes affect cockle burrowing, leading to shell exposure to epibionts colonization and bird predation. Only collaboration between parasitologists and ecologists can reveal the ecological reach, up to humans, of multi‐hosts tapeworms and coccidians using small carnivores as final hosts and their importance «beyond the level of cocktail party anecdotes».

Allusion to cocktail anecdotes prompted afterword author Loreau to state how superficial our knowledge of the ecological impact of parasites still is. He points out that in biomass terms, physics and chemistry laws dictate that production for parasites must be lower than for their hosts. However, they may numerically control dominant hosts via top‐down effects, thus maintaining trophic web basal diversity and recycling. We must change our organism‐centred view of parasitized hosts, and not simply change from viewing them as ‘puppets’, to viewing them as mere ‘vehicles’ for parasite genes. We must view them as ecosystems, in which they play only a structuring role. This is consistent with current trends in systems biology and human microbial ecology. The microbiome being to human health what diversity is to ecosystem stability. Host manipulation is ecological and evolutionary change in action, where the small has invaded the large to become a functional part within it.

In Chapter 10, Poulin and Levri examine potential applications on conservation, agriculture and aquaculture, and animal and human health. They start with a developed picture (Fig. 10.1, p. 174) of how manipulators can affect the outcome of invasion by an exotic species. Parasitic diseases affecting wildlife conservation include rabies, where the virus induces asocial behaviour and elevated aggression in wild carnivores. Fish infection by digenean trematodes causes cataracts reducing host vision and crypsis, making farmed fish easy prey to birds. This shows how animal pathologists and behavioural ecologists would approach a same problem with different conceptions of nature. Vectorborne parasites transmitted by mosquitoes or other flying insects, for example, *Leischmania, Trypanosoma, Plamodium*, are well‐studied cases of great human health importance. Altered behaviour of the insect vectors and/or their hosts can increase transmission at up to eight different nodes in such cycles (Fig. 10.2 p. 178). Toxoplasmosis is an interesting case of vertebrate behaviour manipulation by an intracellular parasite. Its causal agent, *Toxoplasma gondii,* infects 30‐40% of humans worldwide. Unique behavioural effects in rodent hosts support their specific role in boosting the probability of transmission to cats, the natural final hosts. Interestingly, infected humans also exhibit behavioural and psychological effects, despite being unsuitable final hosts for sexual reproduction of the parasite. Personality changes and major psychiatric disorders including congenital schizophrenia have led to much medical and psychological research in the neurology of toxoplasmosis. The authors conclude that to reveal mechanisms involved in ‘accidental’ human infection, we must focus on manipulation‐related traits that have been selected for in the natural rat hosts.

In the afterword Rend and Braithwaite contrast immunologists and behavioural ecologists, in their disciplinal view of what hosts facing behavioural attack must do, that is, kill the manipulator or accommodate it. If vertebrate behaviour has frequently been the target of manipulative parasites, then we expect neural redundancy at the basis of behaviour to maintain functionality under attack. If evolved host traits related to aggregation and promiscuity vs. avoidance and fear are the natural targets, then how behaviour performs without manipulators is potentially revealing. Rend and Braithwaite refer to the hygiene hypothesis which, for human health in general, states that to develop normally, a healthy immunity needs to be stimulated by disease agents. If toxoplasmosis is so common today even in rich human societies as a chronic and more or less benign infection, we have not won the evolutionary race against it as a human parasite. A speculative answer to this paradox leans towards the parasite being on the evolutionarily route to symbiosis (Margulis 1993) with humans. Viewing *T. gondii* as a human symbiont is in agreement with established facts about its epidemiology: 1: high incidence in humans; 2: prevalence‐dependent disorder, that is, it can be tolerated at low levels of brain infection; 3: it is vertically transmitted, that is, humans are ‘dead‐end’ hosts for sexual reproduction, but infected human mothers vertically transmit it to their infants as a congenital disease (Wolf et al. 1939), and so it is trapped to persist benignly in human populations.

In the last chapter, Cézilly and Thomas consider behavioural manipulation outside its mainstream, that is, manipulation by parasites. They evoke human deceitful behaviours as a stimulus to studying these phenomena. In evolutionary terms, they review manipulation in free‐living organisms by considering five categories, from simple sensory exploitation (for example predators using lures to trap prey) to neuroendocrine manipulation, that is, as direct control of behaviour at the source in the brain. They critically examine manipulation, identifying reasons why it can be falsely interpreted as such, unless adaptively explained in terms of benefits to manipulators. Alternatives should always be considered. Despite the greater focus on parasite transmission, most animal interactions involve manipulation, especially intimate and durable interactions. That parasitism involves durable and close contact explains much of its role in behavioural (and physiological) manipulation. Another factor, fitness cost‐benefit ratios in parasitism, is clearly biased against hosts. Costs and benefits in interactions between free‐living organisms (for example confusion, deception, coercion) are not generally unidirectional and invariant as in parasitism and especially predation for which the ‘life‐diner’ analogy is the evolutionary paradigm. Other aspects of manipulation in free‐living species that make them difficult to study include frequency dependence of costs and benefits, variability in consistency and reliability and role reversal affecting possible arms race dynamics between actors.

In the afterword, Kacelnik says that by definition, communication is always manipulative. Evolution favours emitters producing signals (calls, pheromones) that can cause a receiver to act in the emitter's interest. On balance, the receiver's response is positive over the range of natural conditions. To the question if manipulative behaviour in free‐living species is similar to parasite manipulation, Kacelnik leans towards the no answer. Manipulation needs its own theoretical approach for internal parasites. They have direct signalling (hormonal) access to the efferent pathways of host behaviour, in contrast to, for example, bird brood parasites manipulating host parental care, for which it is the host overt behaviours as such that must be targeted.

Much of eukaryotic diversity on earth evolved from the intimate (endosymbiotic) association of microbes that became permanent residents within phagocytic organisms. In metazoan parasitism (as in microbial infection), a ‘*holobiont*’ (Zilber‐Rosenberg and Rosenberg 2008) phenotype is expressed from the interaction of the genome of a parasite (or a pathogen), with that of its host. As the genetic basis of its behaviour, the parasitized host first has its own genome, and then that of the parasite. Seeing the host as the extended phenotype of the parasite (or the reverse) is interesting as a thought exercise, but has limited explanatory power, as parasitized host behaviour cannot be predicted in its full expression. Natural selection has acted jointly on the genome of both the parasite and the host to control behaviour in the parasitized host (Hughes 2013). Elucidating the pathways from genes to phenotypes on each side of a model interaction such as those focused on in this book should help us link the mechanistic basis of host manipulation to its fitness value in the biology of successful parasites.

## Acknowledgement

I thank Pierre Duchesne, Université Laval, for useful comments and suggestions on an earlier version of this review.

## References

[eva12062-bib-0001] Agrawal, A. A. , A. P. Hastings , M. T. J. Johnson , J. L. Maron , and J. ‐A. Salminen 2012 Insect herbivores drive real‐time ecological and evolutionary change in plant populations. Science 338:113–116. 23042894 10.1126/science.1225977

[eva12062-bib-0002] Bohman, B. , L. Jeffares , G. Flematti , R. D. Phillips , K. W. Dixon , R. Peakall and R. A. Barrow . 2012 The discovery of 2‐hydroymethyl‐3 (3‐methyl butyl)‐5‐methylpyrazine: a semiochemical in orchid pollination. Organic Letters 14:2576–2578. 22554451 10.1021/ol300864u

[eva12062-bib-0003] Danks, H. V. 2002 Modification of adverse conditions by insects. Oikos 99:10–24.

[eva12062-bib-0004] Hughes, D. 2013 Pathways to understanding the extended phenotype of parasites in their hosts. The Journal of Experimental Biology 216:142–147. 23225877 10.1242/jeb.077461

[eva12062-bib-0005] Jones, C. G. , J. H. Lawton , and M. Shachak 1997 Positive and negative effects of organisms as physical ecosystem engineers. Ecology 78:1946–1957.

[eva12062-bib-0006] Margulis, L. 1993 Origins of species ‐ acquired genomes and individuality. BioSystems 31:121–125. 8155844 10.1016/0303-2647(93)90039-f

[eva12062-bib-0007] Norbury, C. J. , and I. D. Hickson 2001 Cellular responses to DNA damage. Annual Review of Pharmacology and Toxicology 41:367–401. 10.1146/annurev.pharmtox.41.1.367 11264462

[eva12062-bib-0008] Ponton, F. , F. Otalura‐Luna , T. Lefèvre , P. M. Guérin , C. Lebarbanchon , D. Duneau , D. G. Biron , et al. 2011 Water‐seeking behavior in worm‐infected crickets and reversibility of parasitic manipulation. Behavioral Ecology 22:392–400. 22476265 10.1093/beheco/arq215 PMC3071748

[eva12062-bib-0009] Wheeler, A. W. 1911 The ant colony as an organism. Journal of Morphology 22:307–325.

[eva12062-bib-0010] Wolf, A. , D. Cowen , and B. Paige 1939 Human toxoplasmosis occurrence in infants as an encephalomyelitis verification by transmission to animals. Science 89:226–227. 17737029 10.1126/science.89.2306.226

[eva12062-bib-0011] Zilber‐Rosenberg, I. , and E. Rosenberg 2008 Role of microorganisms in the evolution of animals and plants: the hologenome theory of evolution. FEMS Microbiology Reviews 32:723–735. 18549407 10.1111/j.1574-6976.2008.00123.x

